# Excellent Survival Outcome in a Patient Receiving NALIRIFOX for Metastatic Pancreatic Adenocarcinoma: A Case Report

**DOI:** 10.32604/or.2026.083192

**Published:** 2026-07-16

**Authors:** Abdullah Esmail, Waseem Abdelrahim, Ebtesam Al-Najjar, Raed Zaidan, Tahrir Abdelrahim

**Affiliations:** 1 Section of GI Oncology, Houston Methodist Neal Cancer Center, Houston Methodist Hospital, Houston, TX, USA; 2 Michael E. DeBakey HS for Health Professions, Houston, TX, USA; 3 The University of Houston–Clear Lake, Houston, TX, USA

**Keywords:** NALIRIFOX, long-term survival, pancreatic ductal adenocarcinoma, durable response, case report

## Abstract

**Background:** Pancreatic ductal adenocarcinoma (PDAC) is an aggressive malignancy that is frequently diagnosed at an advanced stage and remains associated with poor survival outcomes. Durable responses to systemic therapy in metastatic disease are uncommon. We report a case of metastatic PDAC with prolonged survival and sustained response following first-line treatment with NALIRIFOX. This report describes a patient with metastatic PDAC who achieved prolonged disease control and sustained response following first-line treatment with NALIRIFOX. **Case Presentation:** A 64-year-old woman presented with abdominal pain, early satiety, weight loss, and markedly elevated CA 19-9 levels. Imaging demonstrated a pancreatic head mass with multiple hepatic metastases, and biopsy confirmed metastatic poorly differentiated PDAC. The patient initiated first-line therapy with NALIRIFOX as part of the NAPOLI-3 protocol and completed 20 cycles over approximately 25 months. Following an early treatment delay secondary to grade 2 neutropenia, she achieved substantial biochemical and radiographic improvement. After eight cycles, imaging demonstrated marked radiographic response with disappearance of the pancreatic lesion and marked regression of hepatic metastases. Disease stability was maintained for more than two years before progression was identified in a dominant hepatic lesion after cycle 20. **Conclusion:** This case highlights the potential for prolonged disease control and extended survival with NALIRIFOX in metastatic PDAC, emphasizing the meaningful clinical benefit that may be achieved in selected patients despite historically poor outcomes.

## 1 Introduction

Pancreatic cancer remains one of the most lethal malignancies, primarily due to late-stage presentation, aggressive metastatic behavior, resistance to chemotherapy, and the limited efficacy of current immunotherapeutic approaches, resulting in a five-year survival rate below 9%. Over the past decades, the incidence of pancreatic cancer has progressively increased across several countries, and it is projected that by 2030 it will become the second leading cause of cancer-related mortality in the United States, the third in Europe, and one of the most common gastrointestinal malignancies worldwide [1,2]. Most pancreatic cancer cases originate from exocrine tissue, with pancreatic ductal adenocarcinoma (PDAC) being the most common histological subtype [3,4,5,6].

Most patients are diagnosed at an advanced or metastatic stage, at which point curative surgical options are no longer feasible [7,8]. Metastatic PDAC is associated with a poor prognosis, with a median overall survival (OS) of approximately 11 months when treated with standard-of-care first-line systemic treatments, including FOLFIRINOX and gemcitabine plus nab-paclitaxel [9,10]. The 5-year survival rate for patients with metastatic disease is as low as 3% [11,12]. Current management relies primarily on combination chemotherapy regimens, including FOLFIRINOX, gemcitabine plus nab-paclitaxel, and liposomal irinotecan-based combinations, with limited durable responses observed in routine practice.

Although advances in molecular profiling, such as next-generation sequencing (NGS), have identified actionable alterations in a subset of patients, and circulating tumor DNA (ctDNA) is being actively investigated as a potential biomarker, these approaches remain investigational and are not yet part of standard clinical management for pancreatic cancer [13,14,15,16,17,18].

However, despite the increasing incidence and the associated poor prognosis, there has been little advancement in novel therapies that have proved to be efficacious, with the standard of care largely remaining unchanged, relying on established chemotherapy drugs with modifications in the dosage or formulation [19,20,21]. There are several reasons for its poor prognosis, including its harsh tumor microenvironment, late clinical presentation, and resistance to conventional chemotherapy agents [22,23]. Furthermore, due to its proximity to surrounding vital vasculature and its propensity for early invasion, a significant majority of local and locally advanced cases are deemed unresectable and, therefore, become incurable with a worse prognosis [24]. NGS and molecular profiling have identified subsets of pancreatic cancer patients with actionable mutations, such as BRCA1/2 or microsatellite instability-high (MSI-H) tumors, which may respond to targeted therapies like PARP inhibitors or immunotherapy, though these remain underutilized in clinical practice [14,15,25]. Despite the advancements in our understanding of cancer biology, there is still a large gap present in our understanding of pancreatic cancer due to its heterogeneous nature. 

Phase 3 NAPOLI-3 trial was a randomized, open-label study comparing NALIRIFOX (liposomal irinotecan, oxaliplatin, leucovorin, and 5-fluorouracil) with gemcitabine plus nab-paclitaxel as first-line therapy in patients with previously untreated metastatic PDAC. The trial demonstrated a statistically significant improvement in OS with NALIRIFOX, establishing it as an alternative first-line treatment option in this setting [26,27,28]. 

Here, we present a case of metastatic poorly differentiated PDAC in a 64-year-old female who achieved prolonged disease control and survival exceeding 30 months following first-line NALIRIFOX therapy. This report describes the clinical course and sustained therapeutic response observed with NALIRIFOX in metastatic PDAC. The case highlights the potential for exceptional clinical benefit in selected patients and underscores the need for further investigation into factors associated with long-term therapeutic response. This report was prepared in accordance with the CARE (CAse REport) guidelines.

According to institutional policy, ethical review and approval were not required for this single-patient case report. Written informed consent was obtained from the patient for publication of this case report and accompanying images. This report was prepared in accordance with the CARE case report guidelines, and the CARE checklist is provided as Supplementary Material S1.

## 2 Case Presentation

A 64-year-old female presented to Houston Methodist Neal Cancer Center with a three-week history of a burning sensation in her epigastric region, which worsened with spicy or fatty foods. She also reported early postprandial fullness, describing the sensation as feeling like a “rock in her stomach.” Additionally, she noted an 8-pound weight loss since her last visit five months ago. The initial physical examination was unremarkable, with a flat abdomen and no abdominal tenderness.

An ultrasound was initially performed, which revealed multiple hypoechoic lesions in the right and left hepatic lobes, measuring 2.3 × 2.7 × 2.9 cm and 2.1 × 1.8 × 1.9 cm, respectively. The common bile duct was dilated, measuring 8 mm, above the normal upper limit. Subsequent magnetic resonance imaging (MRI) and computed tomography (CT) scans showed intra- and extra-hepatic biliary dilation due to a 1.5 × 1.3 cm non-enhancing mass in the pancreatic head, shown in Fig. 1, along with innumerable hepatic cysts and peripheral enhancing solid masses throughout the liver parenchyma, suggestive of metastatic disease. There was no enlargement of the periportal or retroperitoneal lymph nodes. Her family history was notable for ovarian cancer in her mother and throat cancer in her father, with no known family history of pancreatic cancer. She was a never-smoker and reported moderate alcohol consumption.

**Figure 1:**
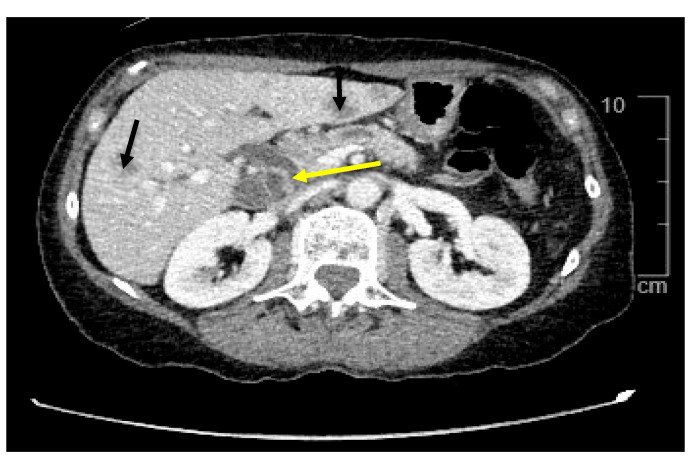
Contrast-enhanced axial CT of the abdomen. The yellow arrow indicates the primary pancreatic head lesion, and the black arrows indicate hepatic metastatic lesions.

An endoscopic ultrasound (EUS) was performed next to further evaluate the suspicious mass and obtain a biopsy. The EUS revealed malignant-appearing, intrinsic, severe stenosis in the second part of the duodenum, leading to gastric outlet obstruction. An irregular mass in the head of the pancreas, measuring 52 mm by 35 mm at its widest diameter, was identified and biopsied using fine-needle aspiration. Multiple hypoechoic lesions were also identified in the right and left lobes of the liver, with the largest lesion measuring 17 mm by 12 mm. The duodenal stenosis was dilated, and a prosthesis was placed a few days later. The patient was subsequently admitted for the placement of internal and external biliary drains, which was performed without complications. Laboratory investigations revealed an elevated CA 19-9 level of 35,924 U/mL. At the time of this measurement, the patient’s total bilirubin had largely normalized to 0.8 mg/dL, suggesting that the CA 19-9 elevation was unlikely to be solely attributable to cholestasis. The neutrophil-to-lymphocyte ratio (NLR) was elevated at 15.1, while the platelet-to-lymphocyte ratio (PLR) was normal at 81.4. A diagnosis of metastatic poorly differentiated PDAC was confirmed through cytologic studies of the biopsies taken. A concise summary of the patient’s clinical presentation, diagnostic findings, and treatment course is provided in Table 1.

**Table 1:** Summary of clinical characteristics and treatment course.

Category	Details
Age/Sex	64-year-old female
Presenting symptoms	Epigastric burning pain, early satiety, postprandial fullness
Weight loss	3.6 kg over 5 months
Diagnosis	Poorly differentiated PDAC
Stage	IV (liver metastases)
Key imaging findings	Pancreatic head mass with biliary dilation and multiple hepatic metastases
Baseline CA 19-9	35,924 U/mL
Molecular testing	Not performed
First-line therapy	NALIRIFOX
Best response	marked radiographic response
CA 19-9 lowest	9 U/mL
Duration of benefit	~25 months
Progression pattern	Dominant hepatic lesion progression
Outcome	Progressed on second-line therapy, hospice care, deceased

Treatment options were discussed with the patient, who elected to receive NALIRIFOX. At treatment initiation, the patient had an ECOG performance status of 0. The patient received NALIRIFOX every 14 days according to the NAPOLI-3 protocol and consisted of liposomal irinotecan 50 mg/m^2^ (73 mg) IV over 90 min, followed by oxaliplatin 60 mg/m^2^ (87.5 mg) IV over 120 min, leucovorin 400 mg/m^2^ (580 mg) IV over 30 min, and continuous-infusion 5-fluorouracil 2400 mg/m^2^ (3480 mg) over 46 h via ambulatory infusion pump.

She tolerated the first cycle well, with only mild constipation and nausea. After the second cycle, she developed grade 2 neutropenia, resulting in a one-week treatment delay. Neutrophil counts fluctuated through treatment; however, hematologic toxicity was generally manageable and did not result in recurrent severe neutropenia or treatment discontinuation. Aside from the early treatment delay, therapy was generally well tolerated throughout the treatment course. The patient experienced mild intermittent fatigue and gastrointestinal symptoms that were managed supportively. No major cumulative toxicities requiring permanent discontinuation of NALIRIFOX were observed during the initial treatment period.

A response evaluation after four cycles of chemotherapy showed stabilization of the pancreatic head mass at 2.3 cm and interval improvement in multifocal hepatic metastatic disease, with the segment 2 lesion decreasing in size from 2.9 × 1.9 cm^2^ to approximately 1.8 × 1.1 cm^2^. The CA 19-9 level also showed significant improvement, decreasing to 2833 U/mL from the initial 35,924 U/mL two months prior.

After eight cycles of therapy and four months since starting treatment, a CT scan showed a further reduction in the size of the metastatic hepatic lesions, consistent with a continued treatment response. The pancreatic head mass was no longer visible on CT. At the six-month follow-up, CT imaging revealed no discrete mass in the pancreas, an atrophic pancreas, and no new hepatic lesions, with a stable right hepatic lobe lesion measuring 9 mm (Fig. 2).

**Figure 2:**
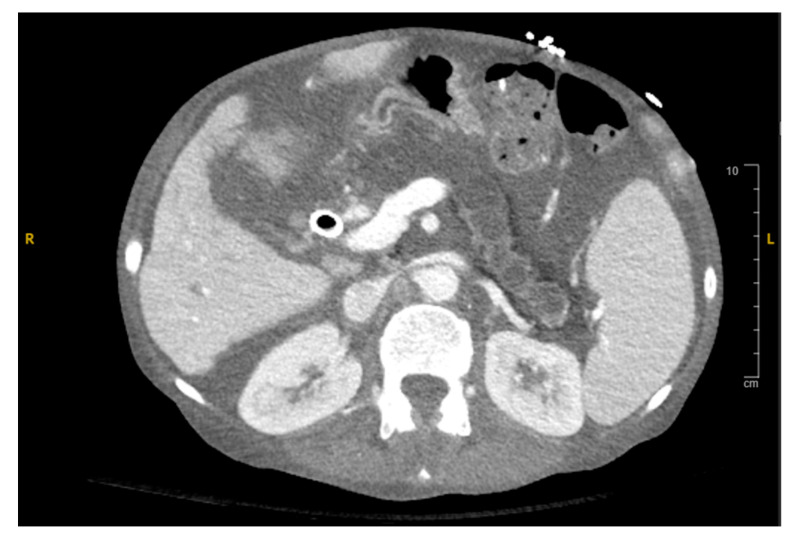
Contrast-enhanced axial CT of the abdomen after treatment demonstrating resolution of the previously identified pancreatic lesion and no new hepatic lesions.

Subsequent imaging continued to demonstrate a stable tumor disease. The patient’s CA 19-9 levels reached a peak low level of 9 U/mL after her 17th cycle, after which it slowly started to increase again (Fig. 3). Imaging done in the following months continued to show a radiographically stable disease. The patient completed 20 cycles of NALIRIFOX over a period of 25 months (Fig. 4). By this point, the CA 19-9 had also risen to 695 U/mL, signaling disease progression. A follow-up CT scan after the 20th cycle indicated disease progression in a dominant liver lesion, which had grown from 0.9 cm to 1.7 cm, in the setting of additional smaller intrahepatic metastatic lesions. Consequently, the patient was switched to second-line therapy with gemcitabine and nab-paclitaxel, initiated with a planned dose reduction. She received two cycles of this regimen, but subsequent imaging showed further disease progression. Following this, the patient’s performance status rapidly declined, marked by significant fatigue. Following her preference, the patient was referred to hospice care and passed away shortly after that. 

**Figure 3:**
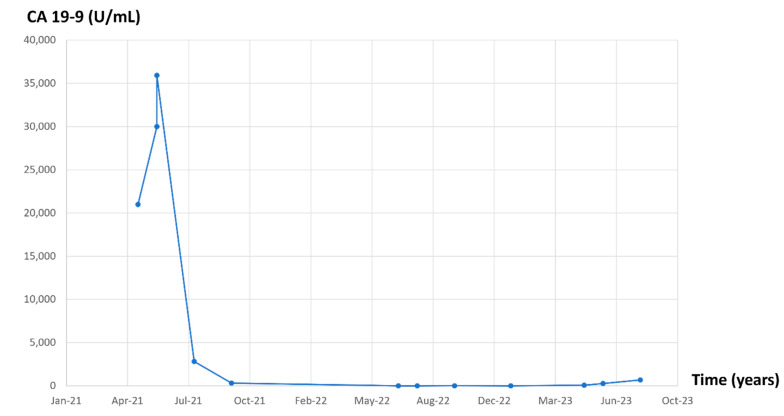
Trend of serum CA 19-9 levels during NALIRIFOX treatment and follow-up. A rapid decline was observed after treatment initiation, followed by a nadir after cycle 17 and gradual increase preceding radiographic disease progression.

**Figure 4:**
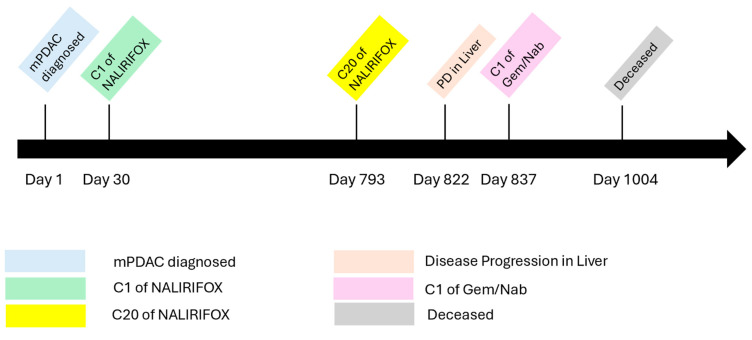
Timeline of disease course, treatment duration, radiographic response, and progression events from diagnosis to death. “C1” indicates Cycle 1 of chemotherapy, and “PD” indicates radiographic disease progression.

## 3 Discussion

Metastatic PDAC is associated with high mortality and morbidity, with limited efficacious chemotherapeutic options. This case presents a prolongation of a patient’s progression-free survival (PFS) and OS using the NALIRIFOX regimen, highlighting the possibility of an exceptional clinical response in selected patients. NALIRIFOX has shown promising results in extending survival in patients with metastatic pancreatic cancer [29]. Nichetti et al. described similar outcomes using NALIRIFOX or FOLFIRINOX, with comparable PFS rates and OS, though with differences in toxicity profiles [30]. Compared with gemcitabine plus nab-paclitaxel, NALIRIFOX demonstrated improved overall survival in the NAPOLI-3 trial, although with increased gastrointestinal and hematologic toxicities [26].

FOLFIRINOX also remains an important first-line regimen for patients with good performance status but is commonly associated with cumulative neuropathy and hematologic adverse effects paclitaxel [9,10]. Therefore, treatment selection should consider patient performance status, comorbidities, toxicity profiles, and treatment goals.

NALIRIFOX is often preferred for patients needing significant tumor shrinkage or those experiencing intolerable neurotoxicity from other treatments. Liposomal irinotecan was designed to improve pharmacokinetic stability and tumor delivery; however, these theoretical advantages have not consistently translated into superior clinical outcomes compared with conventional irinotecan. However, the cost difference between NALIRIFOX and FOLFIRINOX must also be considered in clinical practice, with the cost of liposomal irinotecan 100 times higher than standard irinotecan [31,32]. 

This case demonstrates an OS of more than 30 months, surpassing the median OS of 11.1 months reported in the NAPOLI-3 trial for patients undergoing NALIRIFOX treatment [26]. This significant prolongation could be attributed to differences in performance status, metastatic sites, and primary tumor location. However, an interesting point to note is that several known poor prognostic factors in pancreatic cancer, such as liver metastases, high CA 19-9 levels at diagnosis, and elevated neutrophil to lymphocyte ratio, were present in our patient. Despite these indicators, the patient responded well to treatment, suggesting that additional clinical or biological factors may have contributed to the observed treatment response. These might include specific genetic mutations, variations in drug metabolism, or unique aspects of the tumor microenvironment.

Further research is needed to elucidate these factors. Genomic and molecular analyses of long-term survivors from the NAPOLI-3 trial could reveal novel prognostic factors or genetic markers that might guide treatment decisions and improve patient outcomes. Understanding how NALIRIFOX interacts with the tumor microenvironment and the immune system could also provide valuable insights, as these interactions likely play a role in the observed long-term survival [33,34]. Potential explanations for prolonged response in metastatic PDAC may include homologous recombination repair defects such as BRCA or PALB2 alterations, enhanced platinum sensitivity, variability in irinotecan metabolism, and differences in tumor microenvironment biology. However, these mechanisms remain speculative in the current case because molecular profiling was not performed.

Although NCCN guidelines recommend germline genetic testing for all patients with pancreatic cancer, germline and somatic molecular testing were not performed in this patient. This represents a major limitation of the current report, as the absence of germline and somatic molecular profiling prevents determination of whether the prolonged response was associated with favorable tumor biology or actionable molecular alterations.

Another unique aspect of our case is the reliance on systemic chemotherapy alone to achieve long-term OS benefit, without the need for metastasectomy. Most case reports on long-term survival in metastatic pancreatic cancer highlight the use of multimodal management, including both metastasectomy and systemic chemotherapy [35]. Our patient’s significant survival benefit with NALIRIFOX alone illustrates that prolonged disease control may occur in selected patients treated with systemic therapy alone, expanding treatment options for patients who may not be candidates for surgical intervention.

Another important consideration raised by this case is the distinction between true oligometastatic or oligoprogressive disease and multifocal metastatic progression in pancreatic cancer [36,37]. The concept that metastatic disease is uniformly incurable has been reconsidered in the setting of oligometastatic disease, particularly in selected cancers such as colorectal cancer with isolated liver metastases. In this setting, local treatment of metastatic lesions has been associated with prolonged survival, and several reports have described long-term survival exceeding 10 years following repeated metastasectomy [38,39]. However, the present case does not meet criteria for oligometastatic disease, as imaging demonstrated multiple intrahepatic metastatic lesions rather than a single isolated site of progression, thereby precluding a purely local therapeutic approach. 

This case also raises the question of whether it represents an exceptionally rare outcome or indicates a broader applicability of NALIRIFOX in achieving long-term survival in metastatic pancreatic cancer. This case represents an uncommon and durable response to first-line NALIRIFOX in metastatic PDAC. Although prolonged survival in metastatic pancreatic cancer remains rare, this observation highlights the heterogeneity of treatment response among patients with advanced diseases. Given the single-patient nature of this report, conclusions regarding broader therapeutic applicability should be interpreted cautiously. Further studies are needed to better characterize the clinical and molecular factors associated with exceptional treatment outcomes [26]. This suggests that long-term OS is possible in metastatic pancreatic cancer, reinforcing the need for a deeper understanding of the disease’s basic biology.

## 4 Conclusion

In conclusion, we report a rare case of prolonged survival and durable disease control in a patient with metastatic PDAC treated with first-line NALIRIFOX. This case highlights the possibility of exceptional clinical response in selected patients; however, conclusions regarding broader applicability are limited by the single-patient nature of this report. Further investigation is warranted to better understand the biological and clinical factors associated with long-term response to therapy.

## Data Availability

The data of this case report that supports our results are available on request from the corresponding author, Abdullah Esmail.

## Supplementary Materials

The supplementary material is available online at https://www.techscience.com/doi/10.32604/or.2026.083192/s1.
