# Supplementary material for: Excellent Survival Outcome in a Patient Receiving NALIRIFOX for Metastatic Pancreatic Adenocarcinoma: A Case Report
Source: Oncol Res. 2026 Jul 16;34(8):28. doi: 10.32604/or.2026.083192 (PMC13397356; doi:10.32604/or.2026.083192)
Supplement: Supplementary file 1 [file OncolRes-34-83192-s001.zip › TSP_OR_83192-s001.docx]

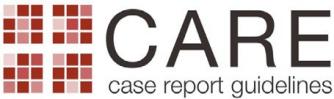

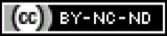

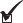
**CARE Checklist of information to include when writing a case report**

| **Topic** | **Item No** | **Checklist item description** | **Reported on Page Number/Line Number** | **Reported on Section/Paragraph** |
| --- | --- | --- | --- | --- |
| Title | 1 | The diagnosis or intervention of primary focus followed by the words “case report” | Page 1, Lines 2–3 | Title |
| Key Words | 2 | 2 to 5 key words that identify diagnoses or interventions in this case report, including "case report" | Page 1, Lines 36–37 | Keywords |
| Abstract  (Structured summary) | 3a | Background: state what is known and unknown; why the case report is unique and what it adds to existing literature. | Page 1, Lines 13–18 | Abstract – Background |
|  | 3b | Case Description: describe the patient’s demographic details, main symptoms, history, important clinical findings, the main diagnosis, interventions, outcomes and follow-ups. | Page 1, Lines 19–28 | Abstract – Case Presentation |
|  | 3c | Conclusions: summarize the main take-away lesson, clinical impact and potential implications. | Page 1, Lines 29–34 | Abstract – Conclusion |
| Introduction | 4 | One or two paragraphs summarizing why this case is unique **(may include references)** | Page 2, Lines 39–95 | Introduction |
| Patient Information | 5a | De-identified patient specific information | Page 3, Lines 97–112 | Case Presentation |
|  | 5b | Primary concerns and symptoms of the patient | Page 3, Lines 97–101 | Case Presentation |
|  | 5c | Medical, family, and psycho-social history including relevant genetic information | Page 3, Lines 109–112 | Case Presentation |
|  | 5d | Relevant past interventions with outcomes | Page 4, Lines 117–130 | Case Presentation |
| Clinical Findings | 6 | Describe significant physical examination (PE) and important clinical findings | Page 3, Lines 97–101 | Case Presentation |
| Timeline | 7 | Historical and current information from this episode of care organized as a timeline | Page 6, Figure 4 | Timeline Figure |
| Diagnostic Assessment | 8a | Diagnostic testing (such as PE, laboratory testing, imaging, surveys). | Page 3, Lines 103–130 | Case Presentation |
|  | 8b | Diagnostic challenges (such as access to testing, financial, or cultural) | NA | NA |
|  | 8c | Diagnosis (including other diagnoses considered) | Page 4, Lines 129–130 | Case Presentation |
|  | 8d | Prognosis (such as staging in oncology) where applicable | Page 4, Table 1 | Case Presentation |
| Therapeutic Intervention | 9a | Types of therapeutic intervention (such as pharmacologic, surgical, preventive, self-care) | Page 4, Lines 134–139 | Case Presentation |
|  | 9b | Administration of therapeutic intervention (such as dosage, strength, duration) | Page 4, Lines 134–139 | Case Presentation |
|  | 9c | Changes in therapeutic intervention (with rationale) | Page 4, Lines 140–147; Page 5, Lines 167–173 | Case Presentation |

| Follow-up and Outcomes | 10a | Clinician and patient-assessed outcomes (if available) | \|  \| \| --- \|  \| Page 5, Lines 162–173 \| \| --- \| | Case Presentation |
| --- | --- | --- | --- | --- | --- | --- |
|  | 10b | Important follow-up diagnostic and other test results | Page 4–5, Lines 148–173 | Case Presentation |
|  | 10c | Intervention adherence and tolerability (How was this assessed?) | Page 4, Lines 140–147 | Case Presentation |
|  | 10d | Adverse and unanticipated events | Page 4, Lines 140–147 | Case Presentation |
| Discussion | 11a | A scientific discussion of the strengths AND limitations associated with this case report | \|  \| \| --- \|  \| Page 7, Lines 226–230 \| \| --- \| | Conclusion |
|  | 11b | Discussion of the relevant medical literature **with references** | Page 6–8, Lines 186–257 | Conclusion |
|  | 11c | The scientific rationale for any conclusions (including assessment of possible causes) | Page 7, Lines 206–225 | Conclusion |
|  | 11d | The primary “take-away” lessons of this case report (without references) in a one paragraph conclusion | Page 8, Lines 259–263 | Conclusion |
| Patient Perspective | 12 | The patient should share their perspective in one to two paragraphs on the treatment(s) they received | NA | NA |
| Informed Consent | 13 | Did the patient give informed consent? Please provide if requested | **Yes**  **✓** | **No** |

*As the checklist was provided upon initial submission, the page number/line number reported may be changed due to copyediting and may not be referable in the published version. In this case, the section/paragraph may be used as an alternative reference.
